# Supplementary material for: Mycorrhizal and endophytic fungi structure forest below-ground symbiosis through contrasting but interdependent assembly processes
Source: Environ Microbiome. 2024 Nov 2;19:84. doi: 10.1186/s40793-024-00628-8 (PMC11531145; doi:10.1186/s40793-024-00628-8)
Supplement: Supplementary file 1 — Supplementary Material 1 [file 40793_2024_628_MOESM1_ESM.pdf]

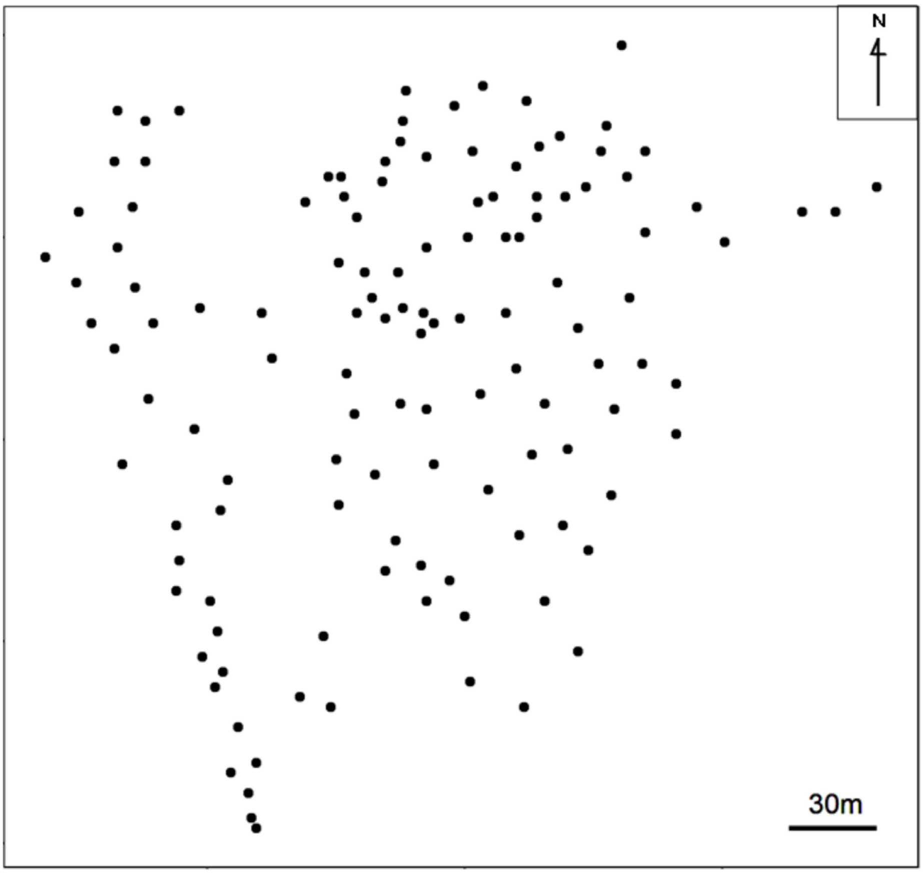

Figure S1：Sampling points

The 126 points where plant roots and soil have been sampled were indicated by dots.

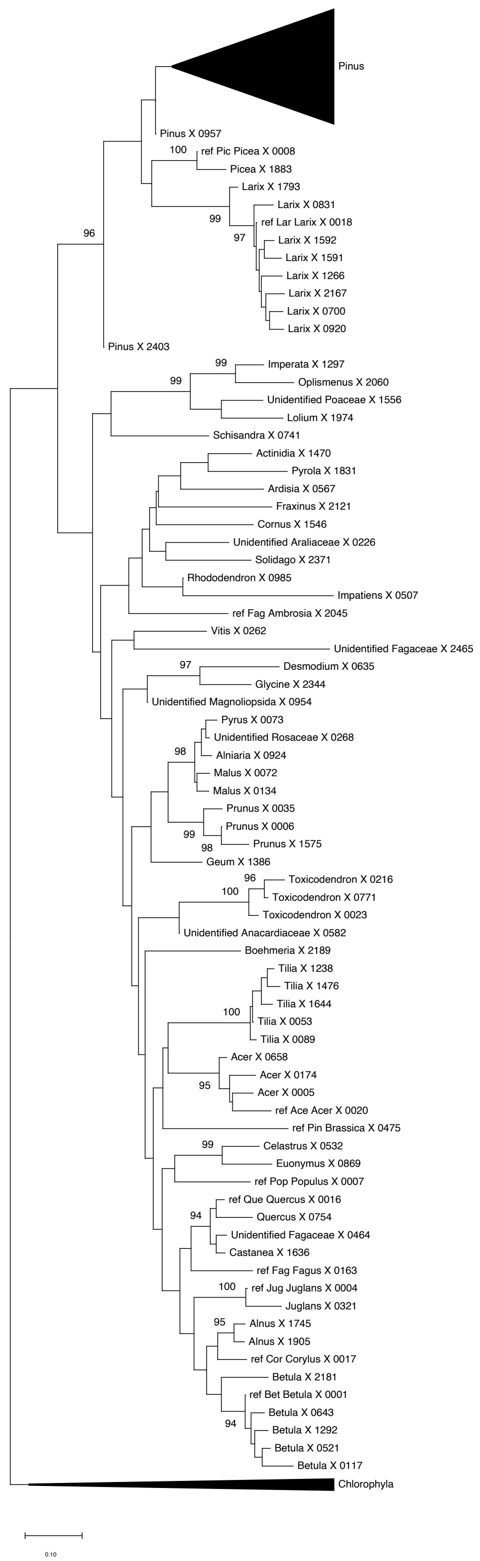

Figure S2: Phylogenetic tree of the host plants. A phylogenetic tree of streptophyta and chlorophyta based on the Neighbor joining method using partial sequences of the plant ITS2 region. Sequences of chlorophyta are grouped together as an outgroup (Chlorophyta). In addition, because a number of homologous sequences identified as *Pinus*, these are also shown as a group (Pinus). For the other sequences, the OTU name and the BLAST identification of the genus are shown at the end of each branch. Sequences derived from reference plant leaf samples were represented as "ref – [Plant name] – [OTU name]". Only on branches with a bootstrap (1000 times) confidence greater than 90% is this confidence indicated.

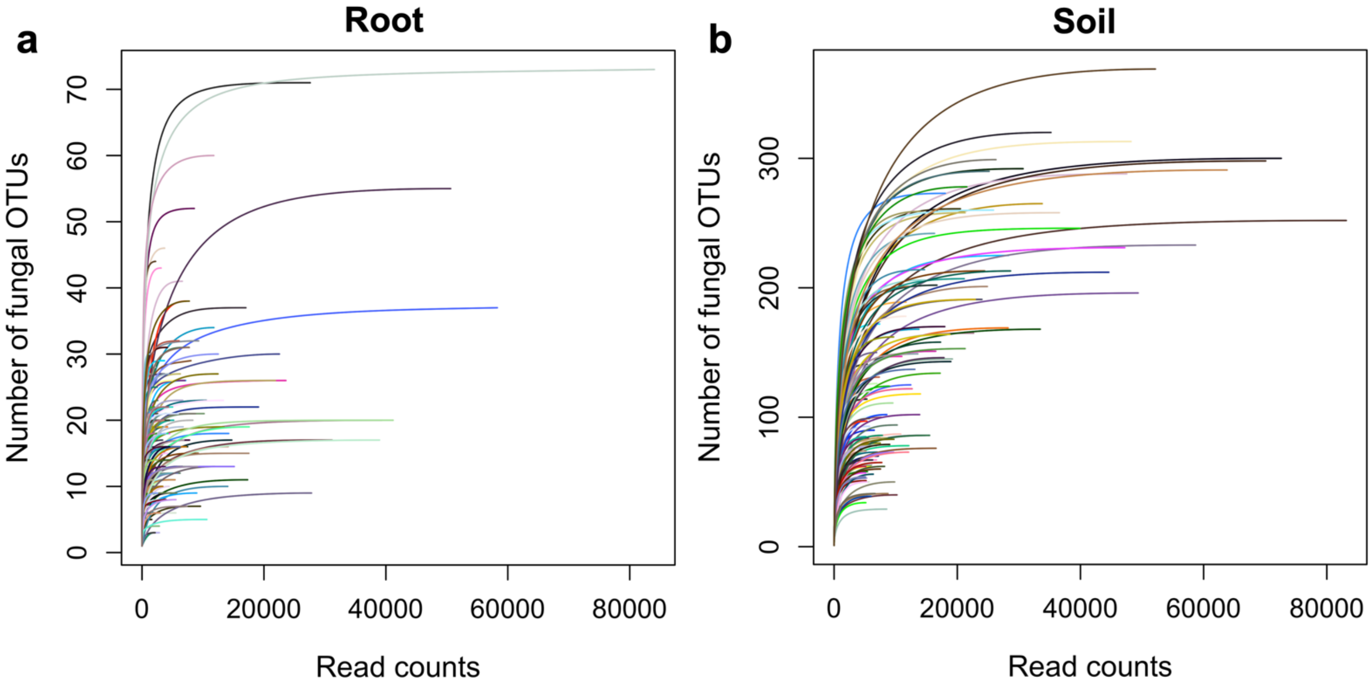

Figure S3: Relationships between the number of sequencing reads and that of fungal OTUs (cutoff sequence similarity: 97%). (a) Number of fungal OTUs detected in plant root samples. For visualization, the relationship between the number of sequencing reads and the number of fungal OTUs detected in 200 randomly selected samples is shown. (b) Number of fungal OTUs detected in soil samples. The relationship between the number of sequencing reads and the number of fungi detected in 124 samples whose reads were successfully obtained.

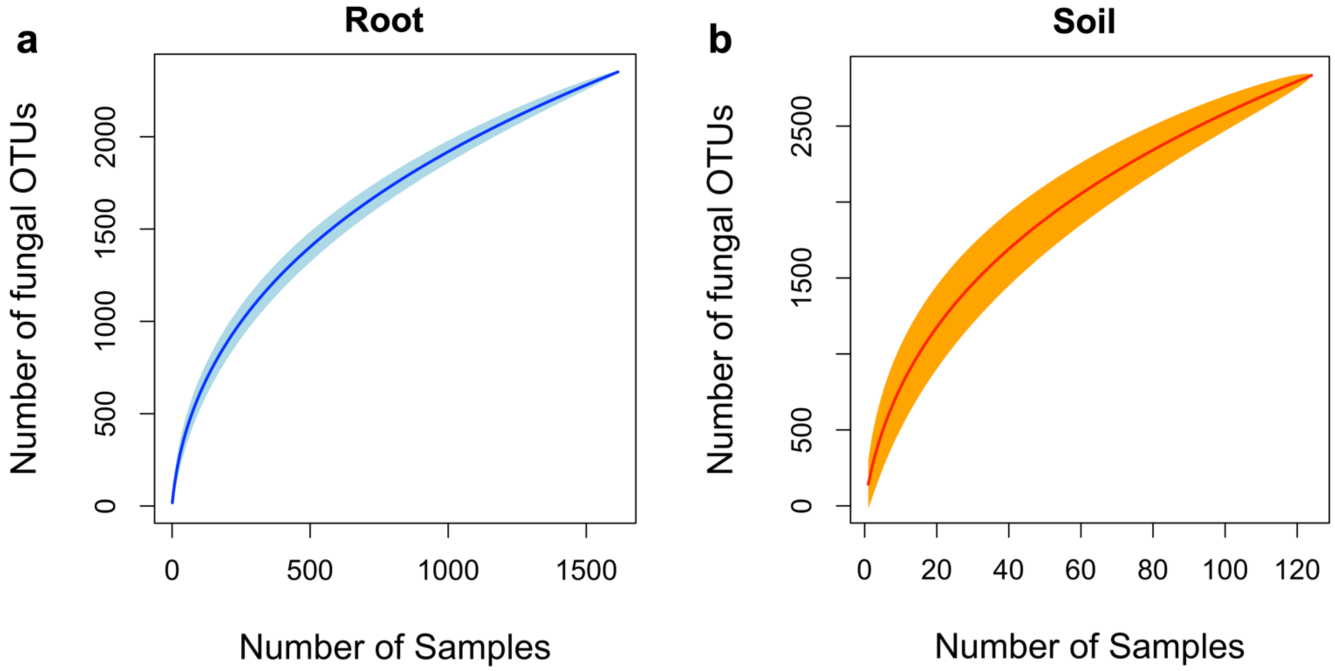

Figure S4: Relationship between the number of root/soil samples and that of fungal OTUs (cutoff sequence similarity: 97%) observed. (a) Number of fungal OTUs in root samples with plant genus identified. In total, sequencing data were successfully obtained from 1615 samples, and 2351 OTUs were detected among samples. (b) Number of fungal OTUs in soil samples. In total, sequencing data were successfully obtained from 124 samples, and 2834 OTUs were detected among samples.

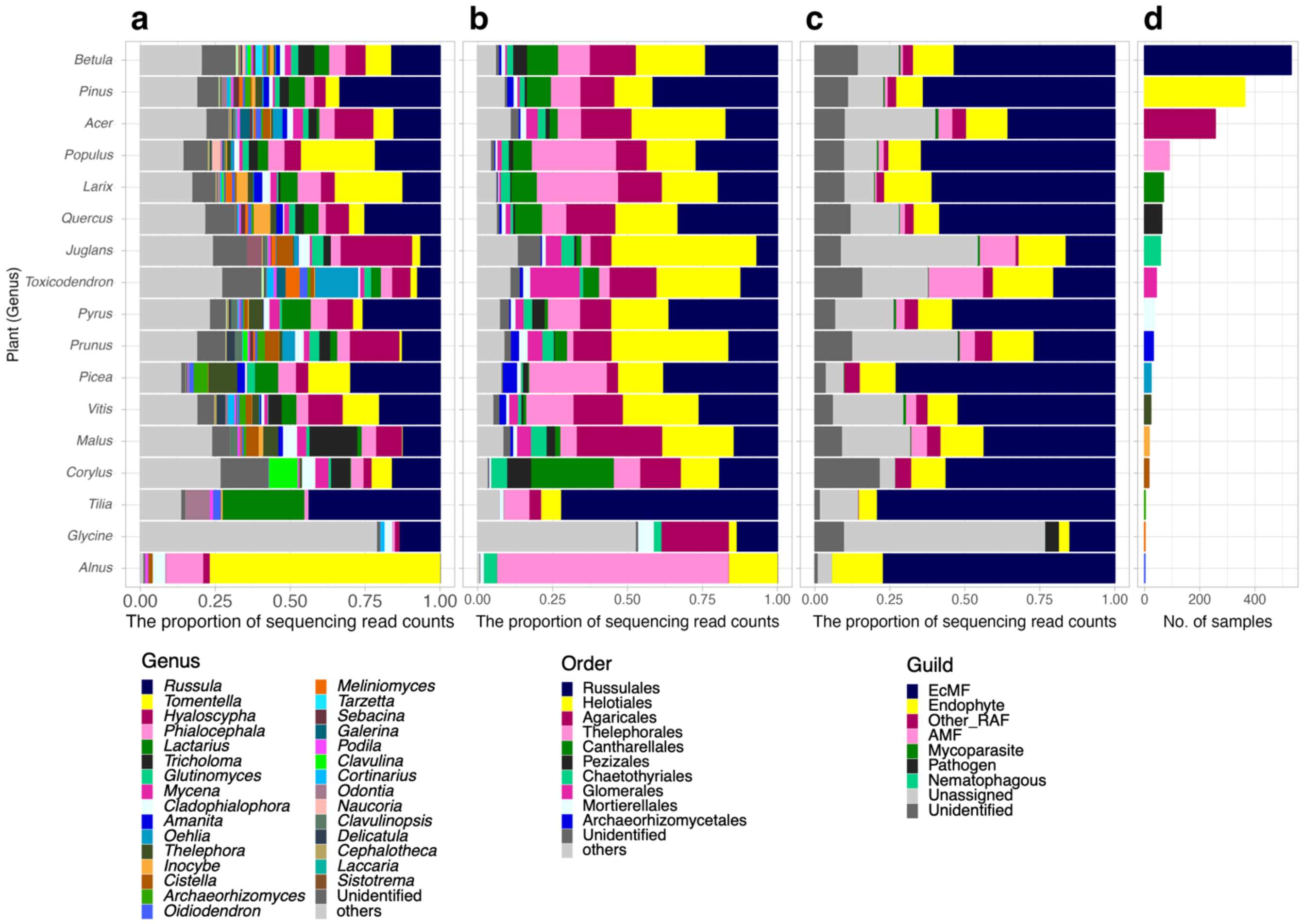

Figure S5: Root fungal community compositions. (a) Genus-level taxonomic compositions. The mean proportions of fungal sequencing reads are shown for each plant genus. (b) Order-level taxonomic compositions. (c) Guild compositions. The guilds (functional groups) of fungal OTUs were inferred with the Fungaltraits database. (d) Number of plant root samples.

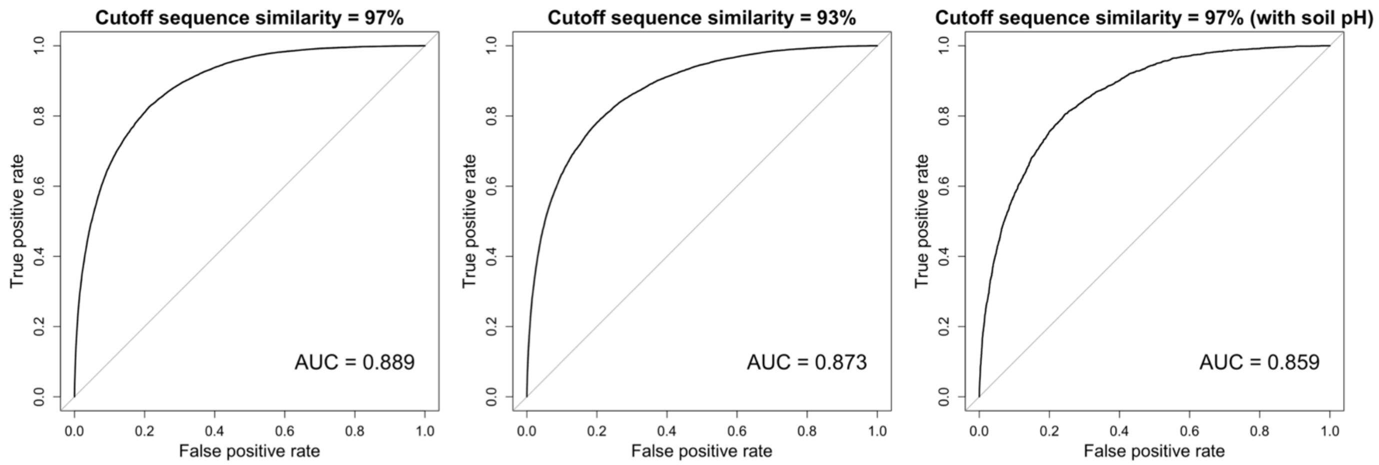

Figure S6: Predictive accuracy of the jSDM models for the distribution of fungal OTU (cutoff sequence similarity: 93%) and fungal OTU (cutoff threshold: 97%) considering the four or five factors. The relationship between the false positive rate and the true positive rate for each model (OTU (93%) and OTU (97%): Model 1 in Table 1, OTU (97%) with soil pH: Model 1 in Table S1) is shown as the prediction accuracy for the distribution of fungal OTU (93%) and fungal OTU (97%). Area under the curve (AUC) is also shown in the figures, respectively.

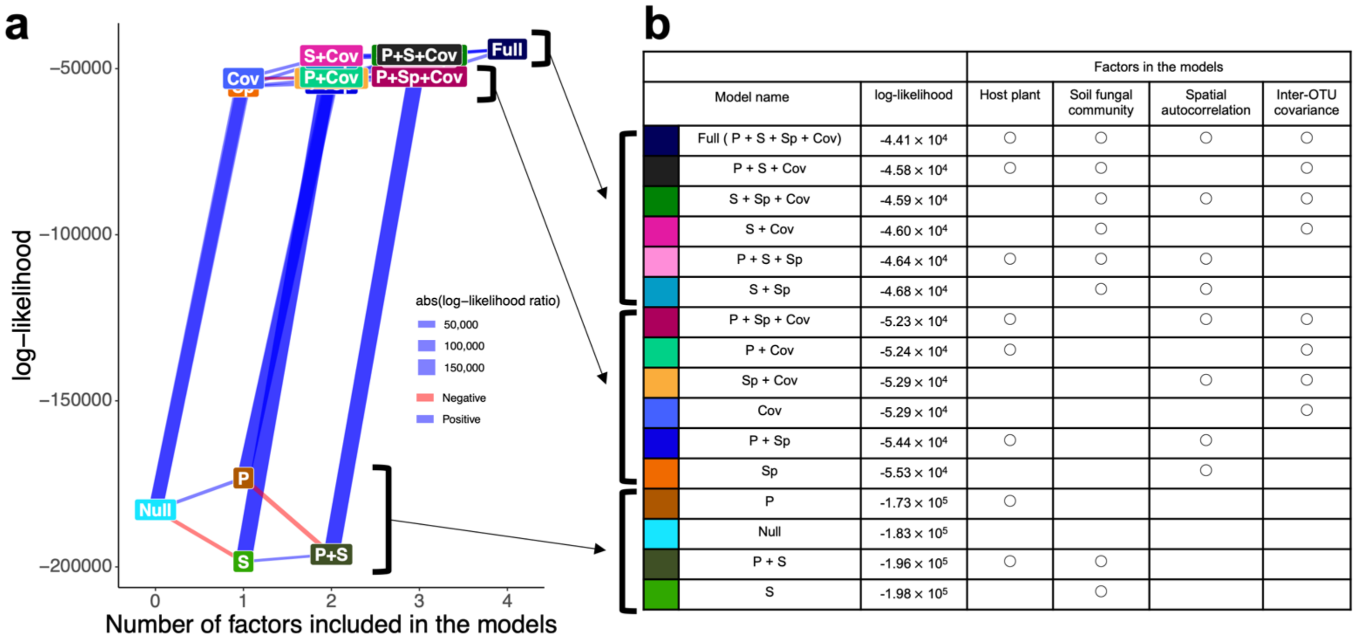

Color	Model name	log-likelihood	Host plant	Soil fungal community	Spatial autocorrelation	Inter-OTU covariance
			Factors in the models			
	Full (P + S + Sp + Cov)	-4.41×10^4	○	○	○	○
	P + S + Cov	-4.58×10^4	○	○		○
	S + Sp + Cov	-4.59×10^4		○	○	○
	S + Cov	-4.60×10^4		○		○
	P + S + Sp	-4.64×10^4	○	○	○	
	S + Sp	-4.68×10^4		○	○	
	P + Sp + Cov	-5.23×10^4	○		○	○
	P + Cov	-5.24×10^4	○			○
	Sp + Cov	-5.29×10^4			○	○
	Cov	-5.29×10^4				○
	P + Sp	-5.44×10^4	○		○	
	Sp	-5.53×10^4			○	
	P	-1.73×10^5	○			
	Null	-1.83×10^5				
	P + S	-1.96×10^5	○	○		
	S	-1.98×10^5		○		

Figure S7: Comparison of the full and partial models in jSDM. (a) Performance of the models. The loglikelihoods of the models (Table 1) are shown. The pairs of full/partial models differing only in the presence/absence of a single explanatory variable are linked with edges, whose thickness represent log-likelihood ratios of the paired models. The OTUs in the input data were defined at the 93%. (b) Models aligned in decreasing order of log-likelihoods. The examined models were roughly classified into three groups in terms of their log-likelihoods.

a

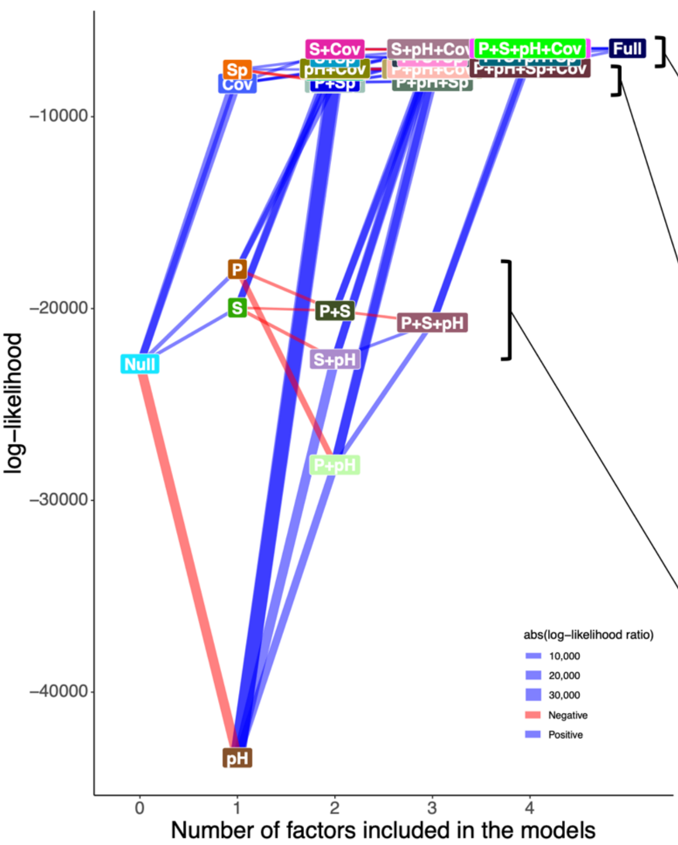

b

	Model name	log-likelihood	Factors in the models: Host plant	Soil fungal community	Soil pH	Spatial autocorrelation	Inter-OTU covariance
	Full (P + S + Sp + Cov)	-6.43×10^4	○	○	○	○	○
	P + S + pH + Cov	-6.44×10^4	○	○	○		○
	S + pH + Sp + Cov	-6.44×10^4		○	○	○	○
	S + pH + Cov	-6.46×10^4		○	○		○
	P + S + Sp + Cov	-6.47×10^4	○	○		○	○
	S + Cov	-6.48×10^4		○			○
	P + S + Cov	-6.48×10^4	○	○			○
	S + Sp + Cov	-6.52×10^4		○		○	○
	P + S + pH + Sp	-6.84×10^4	○	○	○	○	
	P + S + Sp	-6.87×10^4	○	○		○	
	S + pH + Sp	-6.89×10^4		○	○	○	
	S + Sp	-6.91×10^4		○		○	
	P + pH + Sp + Cov	-7.45×10^4	○		○	○	○
	P + pH + Cov	-7.48×10^4	○		○		○
	pH + Cov	-7.48×10^4			○		○
	pH + Sp + Cov	-7.51×10^4			○	○	○
	Sp + Cov	-7.53×10^4				○	○
	P + Sp + Cov	-7.54×10^4	○			○	○
	Sp	-7.54×10^4				○	
	P + Cov	-7.60×10^4	○				○
	P + pH + Sp	-8.15×10^4	○		○	○	
	P + Sp	-8.17×10^4	○			○	
	pH + Sp	-8.24×10^4			○	○	
	Cov	-8.27×10^4					○
	P	-1.80×10^5	○				
	S	-2.00×10^5		○			
	P + S	-2.01×10^5	○	○			
	P + S + pH	-2.07×10^5	○	○	○		
	S + pH	-2.26×10^5		○	○		
	Null	-2.29×10^5					
	P + pH	-2.82×10^5	○		○		
	pH	-4.34×10^5			○		

Figure S8: Comparison of the full and partial models in jSDM with soil pH. (a) Performance of the models. The loglikelihoods of the models (Table 1) are shown. The pairs of full/partial models differing only in the presence/absence of a single explanatory variable are linked with edges, whose thickness represent log-likelihood ratios of the paired models. The OTUs in the input data were defined at the 97%. (b) Models aligned in decreasing order of log-likelihoods. The examined models were roughly classified into three groups in terms of their log-likelihoods.

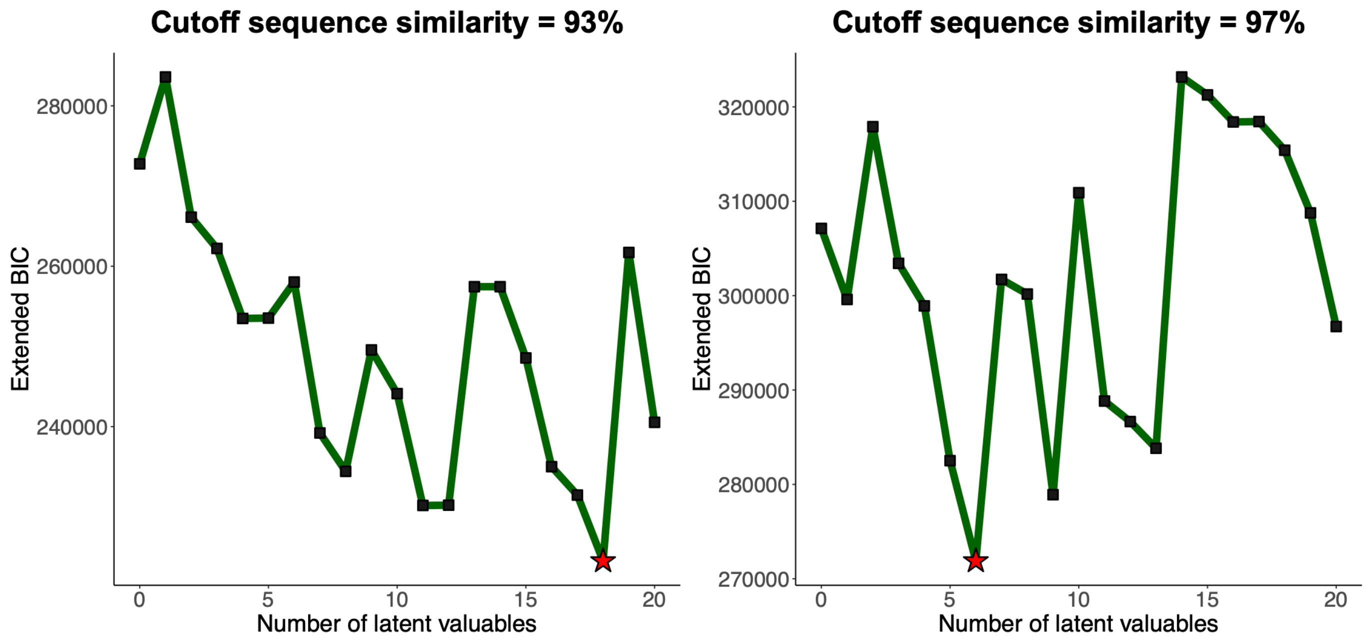

Figure S9: Relationship between the number of assumed latent variables and extended BIC in the co-occurrence network analysis. Co-occurrence network analysis with 0-20 latent variables assumed was conducted for the fungal OTUs included in the analysis in the jSDM analysis. The extended BIC of each estimated network is shown on the vertical axis. The point with the smallest extended BIC is indicated by stars. Results based for the two cutoff sequence similarity thresholds for defining OTUs (93% and 97%) are respectively shown.

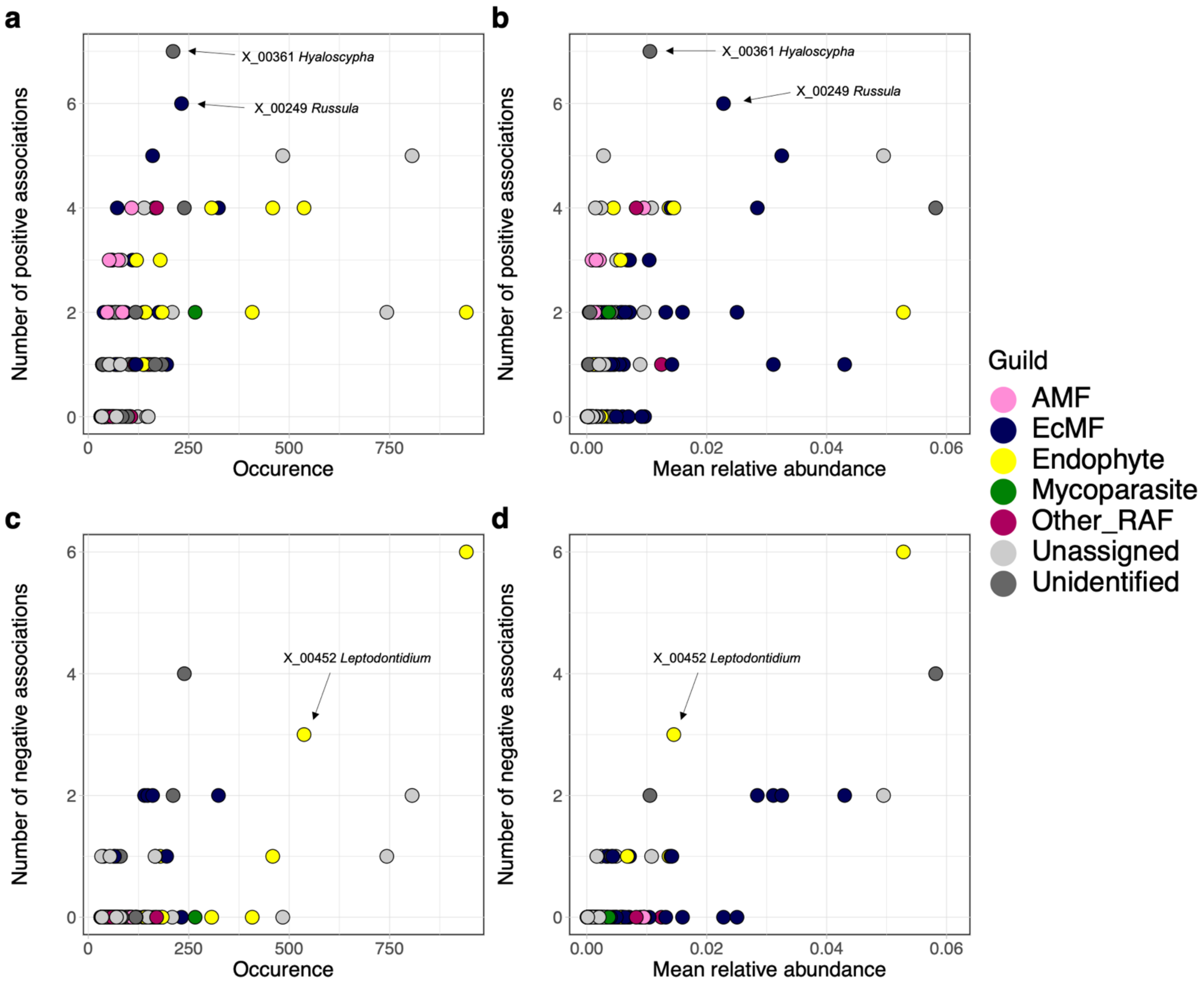

Figure S10: Relationships between fungal assotiation properties and occurrence/abundance of each OTU (cutoff sequence similarity = 97%). Along the vertical axis, the number of positive (a, b) or negative (c, d) associations (i.e., degree centrality) within the SPIEC-EASI networks are shown. Along the horizontal axis, occurrence (a, c) or abundance (b, d) of each OTU is shown. Colors represent fungal functional guilds.

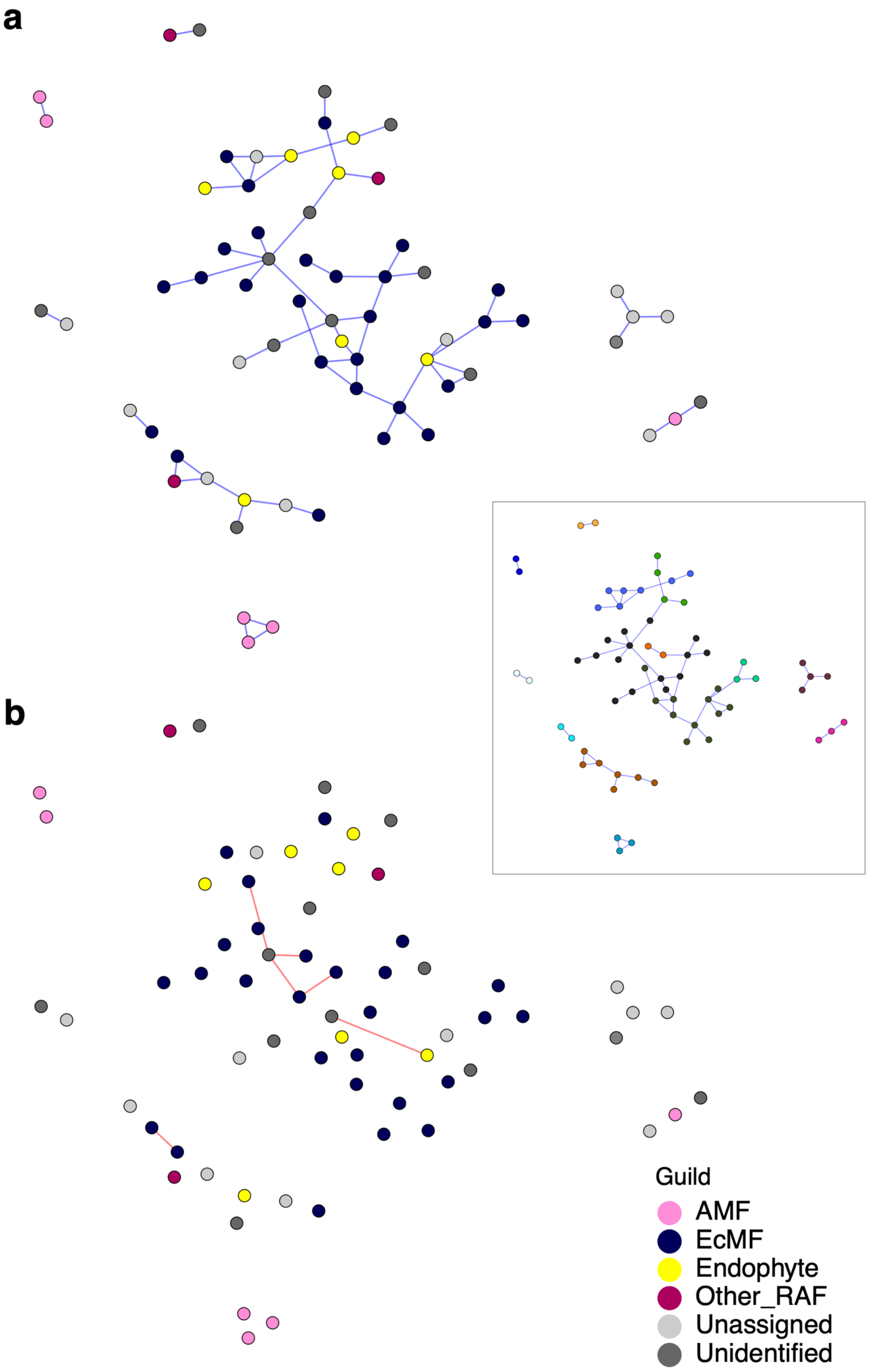
a
b
Guild
AMF
EcMF
Endophyte
Other_RAF
Unassigned
Unidentified

Figure S11: Networks of fungus–fungus associations. Potential interactions between fungal OTUs (defined at the 93% cutoff sequence similarity) were inferred based on the SLR method of SPIEC-EASI. Potential effects of environmental preferences shared between fungal OTUs were controlled in the BIC-selected best model, which included six latent variables. The inferred network architecture was shown separately for positive (a) and negative (b) associations between fungal OTUs. For the positive association network (a), modules of densely associated sets of fungal OTUs were inferred with the Louvain algorithm (see the box in the right side).

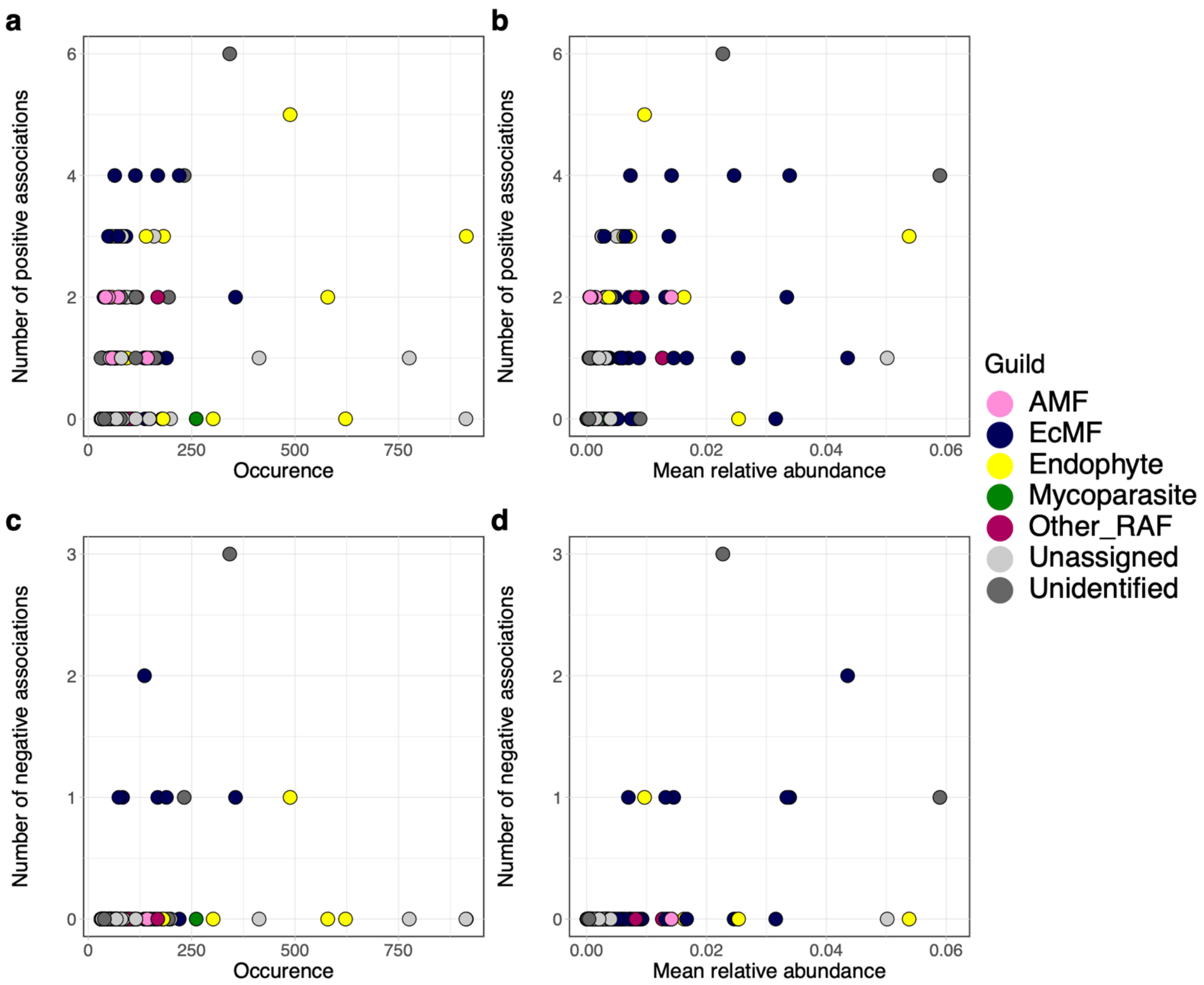

Figure S12: Relationships between fungal assotiation properties and occurrence/abundance of each OTU (cutoff sequence similarity = 93%). Along the vertical axis, the number of positive (a, b) or negative (c, d) associations (i.e., degree centrality) within the SPIEC-EASI networks are shown. Along the horizontal axis, occurrence (a, c) or abundance (b, d) of each OTU is shown. Colors represent fungal functional guilds.

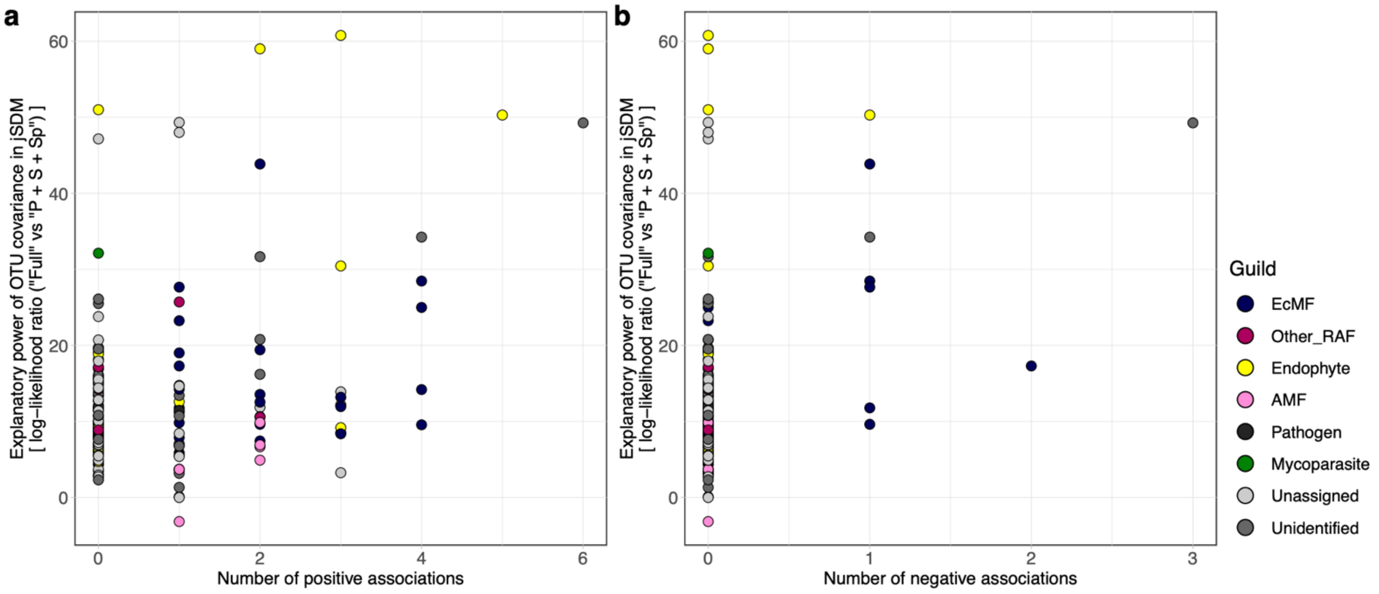

Figure S13: Fungal OTUs (cutoff sequence similarity: 93% similarity) with the strongest signs of species interactions. Fungi potentially playing key roles within the community were explored based on both the jSDM (Fig. 2) and SPIEC-EASI (Fig. 8) frameworks. Based on the jSDM, the log-likelihood ratios of the two models differing in the presence/absence of fungus–fungus covariance [“Full (P + S + SP + Cov)” vs. “P + S + SP”; see Table 1] are shown for fungal OTUs along the vertical axis. Meanwhile, the number of positive (a) or negative (b) associations (i.e., degree centrality) within the SPIEC-EASI networks is shown along the horizontal axis. The degree centrality was significantly correlated with the log-likelihood ratio of the jSDM for both analyses of positive (Kendal’s correlation coefficient = 0.283, $P < 0.001$) and negative (Kendal’s correlation coefficient = 0.256, $P < 0.001$) associations. Colors represent fungal functional guilds. The OTU ID and taxonomy of the OTUs showing the highest levels of log-likelihood ratios or network degree centralities are presented.

Phylum	Class	Order	Family	Genus	Guild (manual)	reference	doi
Basidiomycota	Agaricomycetes	Cantharellales	Hydnaceae	*Sistotrema*	EcMF	Marino et al., 2008	https://doi.org/10.1007/s11557-008-0562-4
Ascomycota	Leotiomycetes	Helotiales	Helotiaceae	*Pseudoclathrosphaerina*	Endophyte	Takashima et al., 2014	https://doi.org/10.11178/jdsa.9.81
Ascomycota	Dothideomycetes	Pleosporales	Pleosporaceae	*Curvularia*	Pathogen	Falloon, 1975	https://doi.org/10.1080/00288233.1976.10426773
Ascomycota	Eurotiomycetes	Chaetothyriales	Herpotrichiellaceae	*Exophiala*	Endophyte	Li et al., 2011	https://doi.org/10.1016/j.scitotenv.2010.12.012
Ascomycota	Leotiomycetes	Helotiales	Leptodontidiaceae	*Leptodontidium*	Endophyte	Upson et al., 2009	https://doi.org/10.1016/j.funeco.2009.02.004
Ascomycota	Sordariomycetes	Diaporthales	Diaporthaceae	*Diaporthe*	Pathogen	Yang et al., 2018	https://doi.org/10.3897/mycokeys.39.26914
Ascomycota	Sordariomycetes	Hypocreales	Nectriaceae	*Fusarium*	Pathogen	Gordon and Martyn, 1997	https://doi.org/10.1146/annurev.phyto.35.1.111

Table S1: Fungal genera whose lifestyle in plant roots were well characterized in previous studies but different functional guilds were inferred with the automatic assignment based on the Fungaltraits database. The table shows their taxonomic information and manually corrected guild with references.

		Factors in the models				
	Model name	Host plant	Soil fungal community	Soil pH	Spatial autocorrelation	inter-OTU covariance
Model 1	Full (P + S + pH + Sp + Cov)	○	○	○	○	○
Model 2	P + S + pH + Sp	○	○	○	○	
Model 3	P + S + pH + Cov	○	○	○		○
Model 4	P + S + Sp + Cov	○	○		○	○
Model 5	P + pH + Sp + Cov	○		○	○	○
Model 6	S + pH + Sp + Cov		○	○	○	○
Model 7	P + S + pH	○	○	○		
Model 8	P + S + Sp	○	○		○	
Model 9	P + S + Cov	○	○			○
Model 10	P + pH + Sp	○		○	○	
Model 11	P + pH + Cov	○		○		○
Model 12	P + Sp + Cov	○			○	○
Model 13	S + pH + Sp		○	○	○	
Model 14	S + pH + Cov		○	○		○
Model 15	S + Sp + Cov		○		○	○
Model 16	pH + Sp + Cov			○	○	○
Model 17	P + S	○	○			
Model 18	P + pH	○		○		
Model 19	P + Sp	○			○	
Model 20	P + Cov	○				○
Model 21	S + pH		○	○		
Model 22	S + Sp		○		○	
Model 23	S + Cov		○			○
Model 24	pH + Sp			○	○	
Model 25	pH + Cov			○		○
Model 26	Sp + Cov				○	○
Model 27	P	○				
Model 28	S		○			
Model 29	pH			○		
Model 30	Sp				○	
Model 31	Cov					○
Model 32	Null					

Table S2: Models examined in the jSDM framework. The explanatory variables examined in each of the full and partial models are presented. The repertoires of the explanatory variables are host plant species (P), the fungal community structure of background soil (S), soil pH (pH), spatial autocorrelation (Sp), and fungus–fungus covariance (co-occurrence) patterns (Cov).

OTU	Log-likelihood ratio	Guild	Scientific Name	Query Cover	E value	Per. Ident	Accession
X_07951	24.3	Unidentified	Venturiales sp.	100%	1.00E-113	98.77%	MW471960.1
X_06373	19.1	AMF	*Rhizophagus* sp.	100%	7.00E-70	93.72%	LC544104.1
X_00149	17.6	Unassigned	Hyaloscyphaceae sp.	100%	1.00E-112	97.96%	MT587170.1
X_00682	17.5	Unassigned	*Hyaloscypha* cf. *hepaticicola*	100%	6.00E-104	95.95%	AY579413.1
X_00128	15.7	Other_RAF	*Archaeorhizomyces borealis*	100%	2.00E-96	100.00%	NR_126144.2
X_00966	13.8	AMF	*Rhizophagus* sp.	100%	6.00E-77	96.32%	LC544104.1
X_09467	13.8	Other_RAF	*Archaeorhizomyces* sp. 'victor nom. seq.'	100%	8.00E-88	97.98%	OQ676542.1
X_01277	13.4	Endophyte	*Pezicula ericae*	100%	1.00E-119	100.00%	MT294405.1
X_06962	13.1	Mycoparasite	fungal sp. 25 GM 18-01	100%	2.00E-96	95.74%	KF359663.1
X_00511	12.6	AMF	*Rhizophagus* sp.	100%	3.00E-67	93.16%	LC544104.1
X_00361	11.2	Unidentified	*Hyaloscypha bicolor*	100%	3.00E-108	96.75%	KX611541.1
X_01506	11.2	AMF	*Rhizophagus* sp.	100%	1.00E-72	94.24%	LC544104.1
X_00301	11.2	Unassigned	Mortierellales sp.	100%	3.00E-113	99.58%	MN385503.1
X_00235	11.1	Endophyte	*Cladophialophora* sp.	100%	4.00E-118	99.59%	MK537116.1
X_03905	10.2	AMF	Glomeraceae sp.	100%	2.00E-77	96.81%	MT765311.1
X_02808	9.71	Unassigned	*Hyaloscypha bicolor*	100%	6.00E-110	97.14%	KX611541.1
X_00467	9.67	Endophyte	*Cladophialophora* sp.	100%	3.00E-113	98.38%	OM744986.1
X_00176	9.35	Endophyte	*Phialocephala fortinii*	100%	1.00E-119	100.00%	MT294419.1
X_00249	7.56	EcMF	*Russula* sp.	100%	2.00E-117	99.59%	OQ421803.1
X_01179	7.48	Endophyte	Helotiaceae sp. II GK-2010	93%	6.00E-104	97.82%	HQ157864.1

Table S3: Effects of host plant identity on fungal OTUs' distribution. For each fungal OTU (cutoff sequence similarity = 97%), impacts of host plants on root-associated fungal distributions was inferred by the log-likelihood ratio between the models with and without the explanatory variable ("P + S + Sp" vs. "S + Sp"; Table 1). The top-20 OTUs with the greatest log-likelihood ratios are shown in the list. For these OTUs, the log-likelihood ratio scores are shown with the OTU IDs, the functional guilds automatically assigned by the Fungaltraits database, and BLAST top-hit results [the scientific name of the matched sequence, query cover, E-value, percent of identity ("Per. Ident"), and NCBI accession number).

OTU	Log-likelihood ratio	Guild	Scientific Name	Query Cover	E value	Per. Ident	Accession
X_00084	1.80×10^2	Unidentified	*Membranomyces* cf. delectabilis GB0090523	80%	4.00E-93	99.49%	JQ638714.1
X_00683	1.66×10^2	Unassigned	*Psathyrella suavissima*	100%	4.00E-100	94.35%	KC992899.1
X_00120	1.64×10^2	EcMF	*Russula* aff. adusta aff. 1 ST-2020	100%	5.00E-98	94.29%	MW024879.1
X_00218	1.49×10^2	EcMF	*Russula* sp.	100%	1.00E-119	100.00%	KU924614.1
X_00249	1.47×10^2	EcMF	*Russula* sp.	100%	2.00E-117	99.59%	OQ421803.1
X_00789	1.46×10^2	EcMF	*Tomentella* sp.	98%	4.00E-100	95.90%	KY686241.1
X_00369	1.34×10^2	EcMF	*Tricholoma* sp. 22 XXD-2021	99%	3.00E-107	97.12%	MW724366.1
X_00265	1.32×10^2	EcMF	*Russula velenovskyi*	100%	1.00E-119	100.00%	AY061721.1
X_00361	1.29×10^2	Unidentified	*Hyaloscypha bicolor*	100%	3.00E-108	96.75%	KX611541.1
X_00625	1.27×10^2	Unassigned	*Leptodontidium* sp.	100%	3.00E-113	98.37%	OM745610.1
X_00686	1.26×10^2	Unassigned	*Mortierella* sp.	100%	6.00E-110	100.00%	ON075228.1
X_00436	1.26×10^2	EcMF	*Russula* sp.	93%	4.00E-106	98.70%	OP133164.1
X_00807	1.24×10^2	EcMF	*Tomentella terrestris*	100%	1.00E-113	98.39%	MW472220.1
X_00282	1.20×10^2	EcMF	*Russula* sp. HMAS 276805	100%	1.00E-100	94.40%	LT602953.1
X_09467	1.20×10^2	Other_RAF	*Archaeorhizomyces* sp. 'victor nom. seq.'	100%	8.00E-88	97.98%	OQ676542.1
X_01102	1.11×10^2	EcMF	*Amanita orientifulva*	97%	5.00E-117	100.00%	LC098745.1
X_18065	1.08×10^2	Unassigned	*Geoglossum simile*	100%	4.00E-105	98.68%	KF854293.1
X_00289	1.05×10^2	EcMF	Thelephoraceae sp. H217	100%	3.00E-94	92.65%	AB634273.1
X_00732	1.03×10^2	EcMF	*Russula* sp.	100%	3.00E-114	98.78%	OQ322556.1
X_00452	1.00×10^2	Endophyte	*Leptodontidium* sp.	100%	1.00E-119	100.00%	OM745579.1

Table S4: Effects of background soil fungal community structure on fungal OTUs' distribution. For each fungal OTU (cutoff sequence similarity = 97%), impacts of background soil community structure on root-associated fungal distributions was inferred by the log-likelihood ratio between the models with and without the explanatory variable ("P + S + Sp" vs. "P + Sp"; Table 1). The top-20 OTUs with the greatest log-likelihood ratios are shown in the list. For these OTUs, the log-likelihood ratio scores are shown with the OTU IDs, the functional guilds automatically assigned by the Fungaltraits database, and BLAST top-hit results [the scientific name of the matched sequence, query cover, E-value, percent of identity ("Per. Ident"), and NCBI accession number).

OTU	Log-likelihood ratio	Guild	Scientific Name	Query Cover	E value	Per. Ident	Accession
X_01617	1.26×10^3	EcMF	*Amphinema* sp. 2 YC-2021	100%	1.00E-119	100.00%	MW546513.1
X_01750	1.24×10^3	Unidentified	fungal sp. 15 GM 14-03	100%	2.00E-65	90.45%	KF359653.1
X_00741	1.21×10^3	EcMF	*Laccaria* sp. 'PA01'	100%	4.00E-118	99.59%	OP749048.1
X_01704	1.21×10^3	Unassigned	*Ramariopsis* sp.	100%	5.00E-111	97.56%	MT644620.1
X_02651	1.20×10^3	Unidentified	Ascomycota sp.	100%	3.00E-114	98.78%	OM744898.1
X_04225	1.20×10^3	Unidentified	*Ramicandelaber taiwanensis*	25%	5.00E-16	95.16%	NR_165200.1
X_01329	1.20×10^3	Unidentified	*Clavulinopsis luteoalba*	100%	2.00E-79	90.28%	OP538704.1
X_11552	1.19×10^3	Unidentified	*Glomus macrocarpum*	100%	3.00E-80	97.33%	LC544108.1
X_01327	1.19×10^3	Unidentified	fungal sp.	100%	1.00E-99	94.29%	MW471758.1
X_00739	1.19×10^3	EcMF	*Naucoria bohemica*	100%	3.00E-114	98.78%	MW243069.1
X_00883	1.18×10^3	Unassigned	*Galerina* sp.	100%	1.00E-93	92.24%	OK161020.1
X_01353	1.18×10^3	EcMF	*Tomentella* sp. 280 VM-2015	100%	5.00E-117	99.18%	KP783478.1
X_02352	1.18×10^3	Unassigned	*Ramariopsis avellaneo-inversa*	100%	1.00E-112	98.37%	MT055954.1
X_01875	1.18×10^3	EcMF	*Sebacina* sp.	96%	2.00E-84	90.79%	OQ410802.1
X_00880	1.17×10^3	EcMF	*Russula burlinghamiae*	88%	4.00E-99	98.61%	MZ157246.1
X_01821	1.16×10^3	Unidentified	Helotiales sp.	100%	5.00E-111	98.33%	OM745587.1
X_00030	1.15×10^3	EcMF	*Tarzetta catinus*	100%	9.00E-114	100.00%	LC619231.1
X_04800	1.15×10^3	Unidentified	Mortierellales sp. RB-2011	100%	4.00E-99	95.18%	JQ272348.1
X_00289	1.15×10^3	EcMF	Thelephoraceae sp. H217	100%	3.00E-94	92.65%	AB634273.1
X_00718	1.14×10^3	Unidentified	Sordariomycetes sp. SM13-17-5-3	100%	3.00E-39	98.06%	MN905840.1

Table S5: Effects of spatial autocorrelation on fungal OTUs' distribution. For each fungal OTU (cutoff sequence similarity = 97%), impacts of spatial autocorrelation on root-associated fungal distributions was inferred by the log-likelihood ratio between the models with and without the explanatory variable ("P + S + Sp" vs. "P + S"; Table 1). The top-20 OTUs with the greatest log-likelihood ratios are shown in the list. For these OTUs, the log-likelihood ratio scores are shown with the OTU IDs, the functional guilds automatically assigned by the Fungaltraits database, and BLAST top-hit results [the scientific name of the matched sequence, query cover, E-value, percent of identity ("Per. Ident"), and NCBI accession number).

OTU	Log-likelihood ratio	Guild	Scientific Name	Query Cover	E value	Per. Ident	Accession
X_00176	50.8	Endophyte	*Phialocephala fortinii*	100%	1.00E-119	100.00%	MT294419.1
X_00452	45.4	Endophyte	*Leptodontidium* sp.	100%	1.00E-119	100.00%	OM745579.1
X_00149	44.9	Unassigned	Hyaloscyphaceae sp.	100%	1.00E-112	97.96%	MT587170.1
X_00235	43.4	Endophyte	*Cladophialophora* sp.	100%	4.00E-118	99.59%	MK537116.1
X_00409	42.0	Endophyte	*Oidiodendron maius*	100%	1.00E-119	100.00%	LC206669.1
X_00054	41.2	Unassigned	*Mortierella* sp. SM13-9-10-3	100%	7.00E-115	100.00%	MN905910.1
X_00301	40.1	Unassigned	Mortierellales sp.	100%	3.00E-113	99.58%	MN385503.1
X_00282	30.8	EcMF	*Russula* sp. HMAS 276805	100%	1.00E-100	94.40%	LT602953.1
X_06962	30.4	Mycoparasite	fungal sp. 25 GM 18-01	100%	2.00E-96	95.74%	KF359663.1
X_00084	26.7	Unidentified	*Membranomyces* cf. delectabilis GB0090523	80%	4.00E-93	99.49%	JQ638714.1
X_00389	26.2	Endophyte	Hyaloscyphaceae sp. CC 14-35	100%	6.00E-110	97.58%	KF359568.1
X_00467	26.2	Endophyte	*Cladophialophora* sp.	100%	3.00E-113	98.38%	OM744986.1
X_00286	24.8	Other_RAF	*Mycena pradensis*	100%	1.00E-119	100.00%	MT196392.1
X_00361	24.1	Unidentified	*Hyaloscypha bicolor*	100%	3.00E-108	96.75%	KX611541.1
X_00900	23.1	Unidentified	Herpotrichiellaceae sp.	100%	1.00E-119	100.00%	OK584496.1
X_00436	22.1	EcMF	*Russula* sp.	93%	4.00E-106	98.70%	OP133164.1
X_01961	21.5	Endophyte	*Cladophialophora chaetospira*	100%	1.00E-119	100.00%	HQ871875.1
X_00249	21.4	EcMF	*Russula* sp.	100%	2.00E-117	99.59%	OQ421803.1
X_00682	20.9	Unassigned	*Hyaloscypha* cf. hepaticicola	100%	6.00E-104	95.95%	AY579413.1
X_01621	20.9	Unassigned	*Mortierella* sp.	100%	6.00E-110	100.00%	MN385496.1

Table S6: Effects of fungus–fungus covariance on fungal OTUs' distribution. For each fungal OTU (cutoff sequence similarity = 97%), impacts of fungus–fungus covariance on root-associated fungal distributions was inferred by the log-likelihood ratio between the models with and without the explanatory variable ("P + S + Sp + Cov" vs. "P + S + Sp"; Table 1). The top-20 OTUs with the greatest log-likelihood ratios are shown in the list. For these OTUs, the log-likelihood ratio scores are shown with the OTU IDs, the functional guilds automatically assigned by the Fungaltraits database, and BLAST top-hit results [the scientific name of the matched sequence, query cover, E-value, percent of identity ("Per. Ident"), and NCBI accession number).

OTU	z-standardized *d'* (fungi)	*P* (FDR)	Guild	Scientific Name	Query Cover	E value	Per. Ident	Accession
X_00966	7.60	*P* < 0.001	AMF	*Rhizophagus sp.*	100%	6.00E-77	96.32%	LC544104.1
X_00511	7.12	*P* < 0.001	AMF	*Rhizophagus sp.*	100%	4.00E-67	93.16%	LC544104.1
X_06373	6.60	*P* < 0.001	AMF	*Rhizophagus sp.*	100%	7.00E-70	93.72%	LC544104.1
X_01506	6.56	*P* < 0.001	AMF	*Rhizophagus sp.*	100%	1.00E-72	94.24%	LC544104.1
X_03905	4.19	*P* < 0.01	AMF	Glomeraceae sp.	100%	2.00E-77	96.81%	MT765311.1
X_02153	4.02	*P* < 0.01	AMF	Glomeraceae sp.	100%	5.00E-78	96.32%	MT765456.1
X_04205	3.10	*P* < 0.05	AMF	Glomeraceae sp.	100%	4.00E-79	95.85%	MT765456.1
X_00700	2.84	*P* < 0.05	EcMF	*Russula emetica*	100%	1.00E-119	100.00%	KX579814.1
X_01277	6.82	*P* < 0.001	Endophyte	*Pezicula ericae*	100%	1.00E-119	100.00%	MT294405.1
X_00176	5.46	*P* < 0.001	Endophyte	*Phialocephala fortinii*	100%	1.00E-119	100.00%	MT294419.1
X_01814	3.47	*P* < 0.05	Endophyte	*Oidiodendron* sp.	100%	8.00E-109	96.73%	OP720876.1
X_00235	3.18	*P* < 0.05	Endophyte	*Cladophialophora* sp.	100%	5.00E-118	99.59%	MK537116.1
X_01179	2.96	*P* < 0.05	Endophyte	Helotiaceae sp. II GK-2010	93%	6.00E-104	97.82%	HQ157864.1
X_06962	4.73	*P* < 0.01	Mycoparasite	fungal sp. 25 GM 18-01	100%	2.00E-96	95.74%	KF359663.1
X_09467	5.34	*P* < 0.001	Other_RAF	*Archaeorhizomyces* sp. 'victor nom. seq.'	100%	9.00E-88	97.98%	OQ676542.1
X_00128	3.73	*P* < 0.01	Other_RAF	*Archaeorhizomyces borealis*	100%	2.00E-96	100.00%	NR_126144.2
X_00682	5.06	*P* < 0.001	Unassigned	*Hyaloscypha* cf. hepaticicola	100%	6.00E-104	95.95%	AY579413.1
X_00301	4.19	*P* < 0.05	Unassigned	*Podila humilis*	100%	4.00E-113	99.58%	JF439486.1
X_01405	4.05	*P* < 0.01	Unassigned	*Dissophora globulifera*	100%	5.00E-118	99.59%	MW393876.1
X_01294	4.00	*P* < 0.01	Unassigned	*Hyaloscypha daedaleae*	100%	1.00E-107	97.14%	MF161321.1
X_09083	3.96	*P* < 0.01	Unassigned	*Chalara angustata*	83%	7.00E-97	100.00%	NR_159786.1
X_00149	3.35	*P* < 0.05	Unassigned	Hyaloscyphaceae sp.	100%	1.00E-112	97.96%	MT587170.1
X_02314	3.33	*P* < 0.05	Unassigned	*Leptodophora orchidicola*	100%	1.00E-119	100.00%	MT294412.1
X_00956	3.08	*P* < 0.05	Unassigned	*Clavulinopsis* sp. 'laeticolor-IN03'	100%	3.00E-114	98.78%	ON650114.1
X_02808	2.95	*P* < 0.05	Unassigned	*Hyaloscypha bicolor*	100%	7.00E-110	97.14%	KX611541.1
X_02874	2.82	*P* < 0.05	Unassigned	Thelephoraceae sp.	96%	1.00E-106	97.46%	OQ410922.1
X_00503	2.76	*P* < 0.05	Unassigned	*Leucoscypha* sp.	82%	4.00E-87	97.03%	OM672930.1
X_07951	8.15	*P* < 0.001	Unidentified	Venturiales sp.	100%	1.00E-113	98.77%	MW471960.1
X_11552	5.70	*P* < 0.001	Unidentified	*Glomus macrocarpum*	100%	3.00E-80	97.33%	LC544108.1
X_04519	3.30	*P* < 0.05	Unidentified	Ascomycota sp.	85%	4.00E-62	89.76%	MN898690.1
X_01585	3.27	*P* < 0.05	Unidentified	*Densocarpa shanorii*	22%	2.00E-16	100.00%	KT361850.1

Table S7: Host preference of fungal OTUs. With randomization test of *d'* metric, fungal OTUs (cutoff sequence similarity = 97%) with significant host preference were screened. Fungal OTUs showing significant host preference patterns are shown with preference metrics (*z*-standardized *d'*), FDR-corrected *P*-values, and BLAST top-hit results [the scientific name of the matched sequence, query cover, E-value, percent of identity ("Per. Ident"), and NCBI accession number).

OTU	Host plant	*2DP* (z-standardized)	*P* (FDR)	Scientific Name	Query Cover	E value	Per. Ident	Accession
X_01277	*Acer*	5.49	*P* < 0.001	*Pezicula ericae*	100%	1.00E-119	100.00%	NR_155653.1
X_06373	*Acer*	4.81	*P* < 0.001	*Rhizophagus* sp.	100%	7.00E-70	93.72%	LC544104.1
X_06962	*Acer*	4.16	*P* < 0.001	fungal sp. 25 GM 18-01	100%	2.00E-96	95.74%	KF359663.1
X_00511	*Acer*	4.08	*P* < 0.001	*Rhizophagus* sp.	100%	3.00E-67	93.16%	LC544104.1
X_09083	*Acer*	4.07	*P* < 0.001	*Chalara angustata*	83%	6.00E-97	100.00%	NR_159786.1
X_07951	*Acer*	-3.75	*P* < 0.001	Venturiales sp.	100%	1.00E-113	98.77%	MW471960.1
X_00682	*Betula*	4.33	*P* < 0.001	*Hyaloscypha* cf. hepaticicola	100%	6.00E-104	95.95%	AY579413.1
X_03905	*Juglans*	4.16	*P* < 0.001	Glomeraceae sp.	100%	2.00E-77	96.81%	MT765311.1
X_04205	*Juglans*	3.90	*P* < 0.001	Glomeraceae sp.	100%	4.00E-79	95.85%	MT765456.1
X_07951	*Pinus*	5.40	*P* < 0.001	Venturiales sp.	100%	1.00E-113	98.77%	MW471960.1
X_09467	*Pinus*	4.61	*P* < 0.001	*Archaeorhizomyces* sp. 'victor nom. seq.'	100%	9.00E-88	97.98%	OQ676542.1
X_02874	*Pinus*	4.26	*P* < 0.01	Thelephoraceae sp.	96%	1.00E-106	97.46%	OQ410922.1
X_00128	*Pinus*	3.93	*P* < 0.001	*Archaeorhizomyces borealis*	100%	2.00E-96	100.00%	NR_126144.2
X_01334	*Pinus*	3.78	*P* < 0.01	*Amphinema* sp. 7 UK-2011	100%	1.00E-119	100.00%	JN943925.1
X_06962	*Pinus*	-3.38	*P* < 0.05	fungal sp. 25 GM 18-01	100%	2.00E-96	95.74%	KF359663.1
X_06373	*Pinus*	-3.82	*P* < 0.001	*Rhizophagus* sp.	100%	7.00E-70	93.72%	LC544104.1
X_00682	*Pinus*	-3.85	*P* < 0.001	*Hyaloscypha* cf. hepaticicola	100%	6.00E-104	95.95%	AY579413.1
X_01506	*Pinus*	-3.90	*P* < 0.001	*Rhizophagus* sp.	100%	1.00E-72	94.24%	LC544104.1
X_00511	*Pinus*	-4.00	*P* < 0.001	*Rhizophagus* sp.	100%	3.00E-67	93.16%	LC544104.1
X_00966	*Pinus*	-4.21	*P* < 0.001	*Rhizophagus* sp.	100%	6.00E-77	96.32%	LC544104.1
X_00149	*Pinus*	-4.35	*P* < 0.001	Hyaloscyphaceae sp.	100%	1.00E-112	97.96%	MT587170.1
X_00807	*Populus*	3.99	*P* < 0.01	*Tomentella terrestris*	100%	1.00E-113	98.39%	MW472220.1
X_00807	*Pyrus*	-4.28	*P* < 0.001	*Tomentella terrestris*	100%	1.00E-113	98.39%	MW472220.1
X_00966	*Toxicodendron*	3.65	*P* < 0.001	*Rhizophagus* sp.	100%	6.00E-77	96.32%	LC544104.1

Table S8: Randomization analysis of plant–fungal associations. Based on a randomization test of two-dimensional preference (*2DP*), pairs of plants and fungal OTUs (cutoff sequence similarity = 97%) showing significant signs of preference/avoidance are screened. The plant–fungus pairs listed are shown with preference metrics (*z*-standardized *2DP*), FDR-corrected *P*-values, and BLAST top-hit results of the fungal OTUs [the scientific name of the matched sequence, query cover, E-value, percent of identity (“Per. Ident”), and NCBI accession number).

OTU	Regression coefficent	*P* (FDR)	Guild	Scientific Name	Query Cover	E value	Per. Ident	Accession
X_06373	0.181	*P* < 0.001	AMF	*Rhizophagus* sp.	100%	7.00E-70	93.72%	LC544104.1
X_01102	0.553	*P* < 0.001	EcMF	*Amanita orientifulva*	97%	5.00E-117	100.00%	LC098745.1
X_00369	0.549	*P* < 0.001	EcMF	*Tricholoma* sp. 22 XXD-2021	99%	3.00E-107	97.12%	MW724366.1
X_01875	0.533	*P* < 0.01	EcMF	*Sebacina* sp.	96%	2.00E-84	90.79%	OQ410802.1
X_01082	0.467	*P* < 0.001	EcMF	*Sistotrema flavorhizomorphae*	100%	1.00E-119	100.00%	NR_178118.1
X_00807	0.462	*P* < 0.001	EcMF	*Tomentella terrestris*	100%	1.00E-113	98.39%	MW472220.1
X_02343	0.452	*P* < 0.001	EcMF	*Cortinarius ferrugineovelatus*	100%	4.00E-118	99.59%	MH930161.1
X_01023	0.421	*P* < 0.001	EcMF	*Sebacina* sp.	96%	8.00E-115	100.00%	OQ410872.1
X_00880	0.415	*P* < 0.001	EcMF	*Russula burlinghamiae*	88%	5.00E-99	98.61%	MZ157246.1
X_00693	0.414	*P* < 0.001	EcMF	*Tomentella* sp. T1228	100%	4.00E-118	99.59%	AB634259.1
X_00218	0.398	*P* < 0.001	EcMF	*Russula vesca*	100%	1.00E-119	100.00%	KX094999.1
X_00766	0.384	*P* < 0.001	EcMF	*Tuber* sp.	83%	1.00E-94	99.02%	LC556138.1
X_00700	0.357	*P* < 0.001	EcMF	*Russula emetica*	100%	1.00E-119	100.00%	KX579814.1
X_01551	0.349	*P* < 0.001	EcMF	*Tomentella* sp.	100%	1.00E-112	97.96%	OQ418543.1
X_00436	0.340	*P* < 0.001	EcMF	*Russula* sp.	93%	4.00E-106	98.70%	OP133164.1
X_00337	0.332	*P* < 0.001	EcMF	*Lactarius vinaceorufescens*	99%	4.00E-112	98.35%	OP749847.1
X_01649	0.315	*P* < 0.001	EcMF	*Inocybe suaveolens*	100%	5.00E-117	99.18%	HQ604205.1
X_01134	0.311	*P* < 0.001	EcMF	*Clavulina amethystina*	100%	3.00E-107	96.33%	MN959776.1
X_00440	0.303	*P* < 0.001	EcMF	*Russula turci*	100%	1.00E-119	100.00%	KF002780.1
X_00888	0.301	*P* < 0.001	EcMF	*Tomentella subtestacea*	100%	8.00E-109	96.73%	MW546519.1
X_00249	0.295	*P* < 0.001	EcMF	*Russula* sp.	100%	2.00E-117	99.59%	OQ421803.1
X_01210	0.294	*P* < 0.001	EcMF	*Pachyphlodes* sp.	100%	5.00E-118	99.59%	OM672915.1
X_00800	0.288	*P* < 0.01	EcMF	*Tomentella sublilacina*	100%	1.00E-119	100.00%	AJ889976.1
X_00030	0.285	*P* < 0.001	EcMF	*Tarzetta catinus*	100%	9.00E-114	100.00%	LC619231.1
X_00120	0.267	*P* < 0.001	EcMF	*Russula* aff. adusta aff. 1 ST-2020	100%	5.00E-98	94.29%	MW024879.1
X_00789	0.257	*P* < 0.001	EcMF	*Tomentella* sp.	98%	4.00E-100	95.90%	KY686241.1
X_02666	0.247	*P* < 0.01	EcMF	*Russula* sp. HMAS 276805	100%	1.00E-105	96.34%	LT602953.1
X_01361	0.245	*P* < 0.05	EcMF	*Laccaria bicolor*	100%	6.00E-117	99.18%	JX504116.1
X_01617	0.236	*P* < 0.001	EcMF	*Amphinema* sp. 2 YC-2021	100%	1.00E-119	100.00%	MW546513.1
X_00282	0.236	*P* < 0.001	EcMF	*Russula* sp. HMAS 276805	100%	1.00E-100	94.40%	LT602953.1
X_00226	0.133	*P* < 0.01	EcMF	*Lactarius tabidus*	100%	1.00E-119	100.00%	KX095062.1
X_01825	0.488	*P* < 0.001	Endophyte	Helotiaceae sp. 3 GM 13-01	100%	1.00E-112	97.96%	KF359565.1
X_00862	0.434	*P* < 0.001	Endophyte	Helotiaceae sp. 3 GM 13-01	100%	8.00E-96	93.47%	KF359565.1
X_00723	0.228	*P* < 0.001	Endophyte	*Oidiodendron* sp.	100%	1.00E-119	100.00%	OM745603.1
X_01179	0.197	*P* < 0.05	Endophyte	Helotiaceae sp. II GK-2010	93%	6.00E-104	97.82%	HQ157864.1
X_00235	0.180	*P* < 0.001	Endophyte	*Cladophialophora chaetospira*	100%	4.00E-118	99.59%	KF359558.1
X_00389	0.167	*P* < 0.01	Endophyte	Hyaloscyphaceae sp. CC 14-35	100%	7.00E-110	97.58%	KF359568.1
X_00452	0.106	*P* < 0.01	Endophyte	*Leptodontidium* sp.	100%	1.00E-119	100.00%	OM745579.1
X_09467	0.290	*P* < 0.001	Other_RAF	*Archaeorhizomyces* sp. 'victor nom. seq.'	100%	9.00E-88	97.98%	OQ676542.1
X_01025	0.276	*P* < 0.05	Other_RAF	*Mortierella* sp. SM13-27-18-1	100%	7.00E-109	100.00%	MN905904.1
X_00286	0.239	*P* < 0.001	Other_RAF	*Mycena pradensis*	100%	1.00E-119	100.00%	MT196392.1
X_00128	0.157	*P* < 0.01	Other_RAF	*Archaeorhizomyces borealis*	100%	2.00E-96	100.00%	NR_126144.2
X_00860	0.574	*P* < 0.001	Unassigned	*Hyaloscypha variabilis*	100%	4.00E-118	99.59%	MN947404.1
X_00956	0.537	*P* < 0.001	Unassigned	*Clavulinopsis* sp. 'laeticolor-IN03'	100%	3.00E-114	98.78%	ON650114.1
X_03252	0.494	*P* < 0.001	Unassigned	*Ramariopsis* sp.	99%	4.00E-112	97.96%	OP896794.1
X_01704	0.470	*P* < 0.001	Unassigned	*Ramariopsis* sp.	100%	5.00E-111	97.56%	MT644620.1
X_03788	0.407	*P* < 0.001	Unassigned	fungal sp.	100%	7.00E-109	97.14%	MF965332.1
X_01204	0.391	*P* < 0.001	Unassigned	*Clavulinopsis* sp. 'laeticolor-IN01'	100%	2.00E-115	98.78%	OR168885.1
X_02874	0.333	*P* < 0.001	Unassigned	Thelephoraceae sp.	96%	1.00E-106	97.46%	OQ410922.1
X_01217	0.321	*P* < 0.001	Unassigned	Clavariaceae sp.	100%	2.00E-104	95.51%	MT587230.1
X_01356	0.304	*P* < 0.001	Unassigned	*Penicillium montanense*	100%	1.00E-119	100.00%	MT514387.1
X_02352	0.295	*P* < 0.001	Unassigned	*Ramariopsis avellaneo-inversa*	100%	1.00E-112	98.37%	MT055954.1
X_18065	0.294	*P* < 0.001	Unassigned	*Geoglossum simile*	100%	5.00E-105	98.68%	KF854293.1
X_03539	0.270	*P* < 0.001	Unassigned	*Odontia parvispina*	100%	5.00E-92	90.51%	MW033328.1
X_01807	0.259	*P* < 0.05	Unassigned	*Entoloma* aff. rhodocylix	100%	5.00E-118	99.59%	ON943248.1
X_00625	0.246	*P* < 0.001	Unassigned	*Leptodontidium* sp.	100%	3.00E-113	98.37%	OM745610.1
X_01294	0.235	*P* < 0.001	Unassigned	*Hyaloscypha daedaleae*	100%	1.00E-107	97.14%	MF161321.1
X_00682	0.139	*P* < 0.01	Unassigned	*Hyaloscypha* cf. hepaticicola	100%	6.00E-104	95.95%	AY579413.1
X_00519	0.479	*P* < 0.001	Unidentified	*Aestipascuomyces dupliciliberans*	29%	1.00E-16	98.28%	MW049145.1
X_01750	0.414	*P* < 0.001	Unidentified	fungal sp. 15 GM 14-03	100%	2.00E-65	90.45%	KF359653.1

X_00084	0.404	*P* < 0.001	Unidentified	*Membranomyces* cf. delectabilis GB0090523	80%	4.00E-93	99.49%	JQ638714.1
X_01254	0.389	*P* < 0.001	Unidentified	*Hyaloscypha* sp. ECRU075	100%	5.00E-117	99.18%	KM678388.1
X_02284	0.381	*P* < 0.001	Unidentified	Basidiomycota sp. CC 01-08	84%	2.00E-28	74.01%	KF359631.1
X_01106	0.336	*P* < 0.05	Unidentified	Mortierellales sp. GD22a	100%	7.00E-109	99.56%	EF152530.1
X_02651	0.319	*P* < 0.001	Unidentified	Ascomycota sp.	100%	3.00E-114	98.78%	OM744898.1
X_00166	0.315	*P* < 0.001	Unidentified	Cryptomycota sp.	97%	3.00E-31	76.78%	MT353563.1
X_01819	0.295	*P* < 0.001	Unidentified	fungal sp.	81%	3.00E-57	86.26%	MG190403.1
X_00718	0.290	*P* < 0.001	Unidentified	Sordariomycetes sp. SM13-17-5-3	100%	3.00E-39	98.06%	MN905840.1
X_01585	0.289	*P* < 0.001	Unidentified	*Densocarpa shanorii*	22%	1.00E-16	100.00%	KT361850.1
X_01976	0.279	*P* < 0.001	Unidentified	*Cladophialophora* sp.	100%	5.00E-117	99.59%	MK536958.1
X_00361	0.266	*P* < 0.001	Unidentified	*Hyaloscypha bicolor*	100%	3.00E-108	96.75%	KX611541.1
X_17682	0.246	*P* < 0.01	Unidentified	fungal sp.	82%	1.00E-36	76.47%	MK564906.1
X_01332	0.224	*P* < 0.05	Unidentified	*Russula carmesina*	100%	2.00E-91	92.68%	MT925405.1
X_00211	0.212	*P* < 0.01	Unidentified	Ascomycota sp. RT-2012	100%	4.00E-35	79.60%	JQ711841.1
X_00645	0.204	*P* < 0.01	Unidentified	*Rhizophagus clarus*	100%	1.00E-47	84.92%	FN423697.1
X_01413	0.141	*P* < 0.05	Unidentified	*Rosettozyma petaloides*	100%	9.00E-39	75.61%	NR_174037.1

Table S9: Relationship between fungal OTU occurrence in root and relative abundance in the soil. Fungal OTUs (cutoff sequence similarity = 97%) with significant relationship between occurrence in root samples and relative abundance in soil samples are listed. Fungal OTUs with significant standardized partial regression coefficients in the generalized liner model (GLM) examined in Fig. 6 are shown with FDR-corrected *P*-values, inferred guilds (functional groups), and BLAST top-hit results [the scientific name of the matched sequence, query cover, E-value, percent of identity ("Per. Ident"), and NCBI accession number).

OTU	Number of Co-occurrence	Guild	Scientific Name	Query Cover	E value	Per. Ident	Accession
X_00361	7	Unidentified	*Hyaloscypha bicolor*	100%	3.00E-108	96.75%	KX611541.1
X_00249	6	EcMF	*Russula* sp.	100%	2.00E-117	99.59%	OQ421803.1
X_00054	5	Unassigned	*Mortierella* sp. SM13-9-10-3	100%	7.00E-115	100.00%	MN905910.1
X_00149	5	Unassigned	Hyaloscyphaceae sp.	100%	1.00E-112	97.96%	MT587170.1
X_00226	5	EcMF	*Lactarius tabidus*	100%	1.00E-119	100.00%	MH862189.1
X_00084	4	Unidentified	*Membranomyces* cf. delectabilis GB0090523	80%	4.00E-93	99.49%	JQ638714.1
X_00235	4	Endophyte	*Cladophialophora chaetospira*	100%	5.00E-118	99.59%	KF359558.1
X_00265	4	EcMF	*Russula velenovskyi*	100%	1.00E-119	100.00%	AY061721.1
X_00282	4	EcMF	*Russula* sp. HMAS 276805	100%	1.00E-100	94.40%	LT602953.1
X_00452	4	Endophyte	*Leptodontidium* sp.	100%	1.00E-119	100.00%	OM745579.1
X_00467	4	Endophyte	*Cladophialophora* sp. L3-3-NN-2016	100%	4.00E-113	98.38%	MN148682.1
X_00625	4	Unassigned	*Leptodontidium* sp.	100%	4.00E-113	98.37%	OM745610.1
X_00683	4	Unassigned	*Psathyrella suavissima*	100%	4.00E-100	94.35%	KC992899.1
X_00686	4	Unassigned	*Mortierella* sp.	100%	6.00E-110	100.00%	ON075228.1
X_06373	4	AMF	*Rhizophagus* sp.	100%	7.00E-70	93.72%	LC544104.1
X_09467	4	Other_RAF	*Archaeorhizomyces* sp. 'victor nom. seq.'	100%	9.00E-88	97.98%	OQ676542.1
X_00389	3	Endophyte	Hyaloscyphaceae sp. CC 14-35	100%	7.00E-110	97.58%	KF359568.1
X_00511	3	AMF	*Rhizophagus* sp.	100%	4.00E-67	93.16%	LC544104.1
X_00693	3	EcMF	*Tomentella* sp. T1228	100%	5.00E-118	99.59%	AB634259.1
X_00732	3	EcMF	*Russula* sp.	100%	3.00E-114	98.78%	OQ322556.1

Table S10: Degree centrality of fungal OTUs (cutoff sequence similarity = 97%) in the positive fungus–fungus association network. With the SLR method of SPIEC-EASI analysis, fungus–fungus associations were inferred by controlling the potential effects of shared environmental preference (Fig. S10a, b). Top-20 fungal OTUs with the highest degree centrality scores (i.e., the number of network links) within the positive association network are shown with the information of inferred guilds (functional groups) and BLAST top-hit results [the scientific name of the matched sequence, query cover, E-value, percent of identity ("Per. Ident"), and NCBI accession number).

OTU	Number of associations	Guild	Scientific Name	Query Cover	E value	Per. Ident	Accession
X_00176	6	Endophyte	*Phialocephala fortinii*	100%	1.00E-119	100.00%	MT294419.1
X_00084	4	Unidentified	*Membranomyces* cf. delectabilis GB0090523	80%	4.00E-93	99.49%	JQ638714.1
X_00452	3	Endophyte	*Leptodontidium* sp.	100%	1.00E-119	100.00%	OM745579.1
X_00120	2	EcMF	*Russula* aff. adusta aff. 1 ST-2020	100%	6.00E-98	94.29%	MW024879.1
X_00149	2	Unassigned	Hyaloscyphaceae sp.	100%	1.00E-112	97.96%	MT587170.1
X_00218	2	EcMF	*Russula vesca*	100%	1.00E-119	100.00%	KX094999.1
X_00226	2	EcMF	*Lactarius tabidus*	100%	1.00E-119	100.00%	KX095062.1
X_00282	2	EcMF	*Russula* sp. HMAS 276805	100%	1.00E-100	94.40%	LT602953.1
X_00361	2	Unidentified	*Hyaloscypha bicolor*	100%	3.00E-108	96.75%	KX611541.1
X_00030	1	EcMF	*Tarzetta catinus*	100%	1.00E-113	100.00%	LC619231.1
X_00235	1	Endophyte	*Cladophialophora* sp.	100%	5.00E-118	99.59%	MK537116.1
X_00265	1	EcMF	*Russula velenovskyi*	100%	1.00E-119	100.00%	AY061721.1
X_00301	1	Unassigned	*Podila humilis*	100%	4.00E-113	99.58%	JF439486.1
X_00389	1	Endophyte	Hyaloscyphaceae sp. CC 14-35	100%	7.00E-110	97.58%	KF359568.1
X_00436	1	EcMF	*Russula* sp.	93%	4.00E-106	98.70%	OP133164.1
X_00618	1	Unassigned	fungal sp.	100%	5.00E-118	99.59%	MH624582.1
X_00625	1	Unassigned	*Leptodontidium* sp.	100%	4.00E-113	98.37%	OM745610.1
X_00683	1	Unassigned	*Psathyrella suavissima*	100%	4.00E-100	94.35%	KC992899.1
X_01023	1	EcMF	*Sebacina* sp.	96%	9.00E-115	100.00%	OQ410872.1
X_01334	1	Unidentified	*Tylospora* sp.	100%	1.00E-119	100.00%	OR482711.1

Table S11: Degree centrality of the fungal OTUs (cutoff sequence similarity = 97%) in the negative fungus–fungus association network. With the SLR method of SPIEC-EASI analysis, fungus–fungus associations were inferred by controlling the potential effects of shared environmental preference (Fig. S10a, b). Top-20 fungal OTUs with the highest degree centrality scores (i.e., the number of network links) within the negative association network are shown with the information of inferred guilds (functional groups) and BLAST top-hit results [the scientific name of the matched sequence, query cover, E-value, percent of identity ("Per. Ident"), and NCBI accession number).

OTU	Module	Guild	Scientific Name	Query Cover	E value	Per. Ident	Accession
X_02005	1	EcMF	*Russula nigricans*	99%	2.00E-117	99.59%	MW172333.1
X_00739	1	EcMF	*Naucoria bohemica*	100%	3.00E-114	98.78%	MW243069.1
X_00369	1	EcMF	*Tricholoma* sp. 22 XXD-2021	99%	3.00E-107	97.12%	MW724366.1
X_00289	1	EcMF	Thelephoraceae sp. H217	100%	4.00E-94	92.65%	AB634273.1
X_00030	1	EcMF	*Tarzetta catinus*	100%	1.00E-113	100.00%	LC619231.1
X_02874	1	Unassigned	Thelephoraceae sp.	96%	1.00E-106	97.46%	OQ410922.1
X_02808	1	Unassigned	*Hyaloscypha bicolor*	100%	7.00E-110	97.14%	KX611541.1
X_01334	1	Unidentified	*Tylospora* sp.	100%	1.00E-119	100.00%	OR482711.1
X_00361	1	Unidentified	*Hyaloscypha bicolor*	100%	3.00E-108	96.75%	KX611541.1
X_00084	1	Unidentified	*Membranomyces* cf. delectabilis GB0090523	80%	4.00E-93	99.49%	JQ638714.1
X_00218	2	EcMF	*Russula vesca*	100%	1.00E-119	100.00%	KX094999.1
X_01316	2	Other_RAF	Mortierellales sp.	93%	5.00E-111	100.00%	MT533763.1
X_00301	2	Unassigned	*Podila humilis*	100%	4.00E-113	99.58%	JF439486.1
X_00300	2	Unassigned	*Saitozyma podzolica*	100%	7.00E-89	100.00%	KY320605.1
X_00054	2	Unassigned	*Podila minutissima*	100%	8.00E-115	100.00%	MK513846.1
X_00807	3	EcMF	*Tomentella terrestris*	100%	1.00E-113	98.39%	MW472220.1
X_00789	3	EcMF	*Tomentella* sp.	98%	4.00E-100	95.90%	KY686241.1
X_00693	3	EcMF	*Tomentella* sp. T1228	100%	5.00E-118	99.59%	AB634259.1
X_00440	3	EcMF	*Russula turci*	100%	1.00E-119	100.00%	KF002780.1
X_00265	3	EcMF	*Russula velenovskyi*	100%	1.00E-119	100.00%	AY061721.1
X_00120	3	EcMF	*Russula* aff. adusta aff. 1 ST-2020	100%	6.00E-98	94.29%	MW024879.1
X_00235	3	Endophyte	*Cladophialophora* sp.	100%	5.00E-118	99.59%	MK537116.1
X_00682	3	Unassigned	*Hyaloscypha* cf. hepaticicola	100%	6.00E-104	95.95%	AY579413.1
X_00128	4	Other_RAF	*Archaeorhizomyces borealis*	100%	2.00E-96	100.00%	NR_126144.2
X_04645	4	Unassigned	*Pezoloma* sp.	100%	1.00E-106	96.73%	OM951709.1
X_01413	4	Unidentified	*Rosettozyma petaloides*	100%	9.00E-39	75.61%	NR_174037.1
X_00337	5	EcMF	*Lactarius cremicolor*	100%	1.00E-119	100.00%	OR364573.1
X_01277	5	Endophyte	*Pezicula ericae*	100%	1.00E-119	100.00%	MT294405.1
X_00452	5	Endophyte	*Leptodontidium* sp.	100%	1.00E-119	100.00%	OM745579.1
X_00176	5	Endophyte	*Phialocephala fortinii*	100%	1.00E-119	100.00%	MT294419.1
X_06962	5	Mycoparasite	fungal sp. 25 GM 18-01	100%	2.00E-96	95.74%	KF359663.1
X_00286	5	Other_RAF	*Mycena pradensis*	100%	1.00E-119	100.00%	MT196392.1
X_01204	5	Unassigned	*Clavulinopsis* sp. 'laeticolor-IN01'	100%	2.00E-115	98.78%	OR168885.1
X_00625	5	Unassigned	*Leptodontidium* sp.	100%	4.00E-113	98.37%	OM745610.1
X_00149	5	Unassigned	Hyaloscyphaceae sp.	100%	1.00E-112	97.96%	MT587170.1
X_01254	5	Unidentified	*Hyaloscypha* sp. ECRU075	100%	6.00E-117	99.18%	KM678388.1
X_00900	5	Unidentified	Herpotrichiellaceae sp.	100%	1.00E-119	100.00%	OK584496.1
X_00211	5	Unidentified	Ascomycota sp. RT-2012	100%	4.00E-35	79.60%	JQ711841.1
X_00732	6	EcMF	*Russula* sp.	100%	3.00E-114	98.78%	OQ322556.1
X_01825	6	Endophyte	Helotiaceae sp. 3 GM 13-01	100%	1.00E-112	97.96%	KF359565.1
X_00862	6	Endophyte	Helotiaceae sp. 3 GM 13-01	100%	9.00E-96	93.47%	KF359565.1
X_09467	6	Other_RAF	*Archaeorhizomyces* sp. 'victor nom. seq.'	100%	9.00E-88	97.98%	OQ676542.1
X_00860	6	Unassigned	*Hyaloscypha variabilis*	100%	5.00E-118	99.59%	MN947404.1
X_00166	6	Unidentified	Cryptomycota sp.	97%	3.00E-31	76.78%	MT353563.1
X_00700	7	EcMF	*Russula emetica*	100%	1.00E-119	100.00%	KX579814.1
X_00226	7	EcMF	*Lactarius tabidus*	100%	1.00E-119	100.00%	KX095062.1
X_00389	7	Endophyte	Hyaloscyphaceae sp. CC 14-35	100%	7.00E-110	97.58%	KF359568.1
X_03607	7	Pathogen	*Fusarium oxysporum*	100%	5.00E-111	100.00%	MF281350.2
X_00686	7	Unassigned	*Mortierella* sp.	100%	6.00E-110	100.00%	ON075228.1
X_00683	7	Unassigned	*Psathyrella suavissima*	100%	4.00E-100	94.35%	KC992899.1
X_00505	7	Unassigned	*Mortierella* sp. S-24	100%	5.00E-104	99.10%	KJ735018.1
X_00503	7	Unassigned	*Leucoscypha* sp.	82%	4.00E-87	97.03%	OM672930.1
X_01649	8	EcMF	*Inocybe umbratica*	100%	6.00E-117	99.18%	OR364571.1
X_01082	8	EcMF	*Sistotrema flavorhizomorphae*	100%	1.00E-119	100.00%	NR_178118.1
X_00249	8	EcMF	*Russula* sp.	100%	2.00E-117	99.59%	OQ421803.1
X_01961	8	Endophyte	*Cladophialophora chaetospira*	100%	1.00E-119	100.00%	HQ871875.1
X_00467	8	Endophyte	*Cladophialophora* sp. L3-3-NN-2016	100%	4.00E-113	98.38%	MN148682.1
X_00409	8	Endophyte	*Oidiodendron maius*	100%	1.00E-119	100.00%	LC206669.1
X_07951	8	Unidentified	Venturiales sp.	100%	1.00E-113	98.77%	MW471960.1
X_01976	8	Unidentified	*Cladophialophora* sp.	100%	6.00E-117	99.59%	MK536958.1
X_02666	9	EcMF	*Russula* sp. HMAS 276805	100%	1.00E-105	96.34%	LT602953.1
X_00436	9	EcMF	*Russula* sp.	93%	4.00E-106	98.70%	OP133164.1
X_00282	9	EcMF	*Russula* sp. HMAS 276805	100%	1.00E-100	94.40%	LT602953.1
X_00723	9	Endophyte	*Oidiodendron* sp.	100%	1.00E-119	100.00%	OM745603.1

X_06373	10	AMF	*Rhizophagus* sp.	100%	7.00E-70	93.72%	LC544104.1
X_01506	10	AMF	*Rhizophagus* sp.	100%	1.00E-72	94.24%	LC544104.1
X_00966	10	AMF	*Rhizophagus* sp.	100%	6.00E-77	96.32%	LC544104.1
X_00511	10	AMF	*Rhizophagus* sp.	100%	4.00E-67	93.16%	LC544104.1
X_18065	10	Unassigned	*Geoglossum simile*	100%	5.00E-105	98.68%	KF854293.1
X_02620	11	Unidentified	*Rhizophagus* sp.	100%	2.00E-45	83.58%	MK418525.1
X_00645	11	Unidentified	*Rhizophagus clarus*	100%	1.00E-47	84.92%	FN423697.1
X_03403	12	EcMF	*Genea hispidula*	100%	2.00E-117	99.59%	MT505214.1
X_02343	12	EcMF	*Cortinarius ferrugineovelatus*	100%	5.00E-118	99.59%	MH930161.1
X_01134	12	EcMF	*Clavulina amethystina*	100%	3.00E-107	96.33%	MN959776.1
X_00894	12	EcMF	*Russula cascadensis*	100%	1.00E-112	97.96%	KF359616.1
X_04205	13	AMF	Glomeraceae sp.	100%	4.00E-79	95.85%	MT765456.1
X_03905	13	AMF	Glomeraceae sp.	100%	2.00E-77	96.81%	MT765311.1
X_02153	13	AMF	Glomeraceae sp.	100%	5.00E-78	96.32%	MT765456.1

Table S12: Modules of densely associated sets of fungal OTUs. The modules of the fungal OTUs (cutoff sequence similarity = 97 %) in the positive co-occurrence network (Fig. 8a) were inferred with the Louvain algorithm. Fungal OTUs are shown with their network modules, inferred guilds (functional groups), and BLAST top-hit results [the scientific name of the matched sequence, query cover, E-value, percent of identity (“Per. Ident”), and NCBI accession number).
